# ﻿*Sedum shunhuangense* (Crassulaceae), a new species from Hunan, China

**DOI:** 10.3897/phytokeys.262.159097

**Published:** 2025-09-12

**Authors:** Zi-Lin Feng, Xiao-Wen Liao, Du Deng, Long-Ping Tang, Shi-Yong Meng, Lei Wu

**Affiliations:** 1 College of Forestry, Central South University of Forestry and Technology, Changsha, 410004, China Central South University of Forestry and Technology Changsha China; 2 College of Life and Environmental Sciences, Hunan University of Arts and Science, Changde, 415000, China Hunan University of Arts and Science Changde China; 3 Hunan Dong’an Shunhuang Mountain National Nature Reserve, Yongzhou, 425901, China Hunan Dong’an Shunhuang Mountain National Nature Reserve Yongzhou China; 4 School of Life Sciences, Peking University, Beijing, 100871, China Peking University Beijing China

**Keywords:** Hunan Province, molecular phylogenetics, morphology, new species, *
Sedum
*

## Abstract

We describe and illustrate *Sedum shunhuangense*, a new species from Hunan, China. The species is morphologically similar to *S. alfredii* and *S. yangjifengensis* but differs in having subequal sepals, papillate seeds, and alternate leaves with caducous lower leaves during flowering. Phylogenetic reconstruction strongly supports two accessions of *S. shunhuangense* as monophyletic and sister to *S. lipingense* and *S. wilsonii*. The species has an estimated wild population of 200 individuals and is assessed as Endangered (EN) under IUCN criteria.

## ﻿Introduction

The Crassulaceae is the most species-rich family within the order Saxifragales and is characterized by succulent leaves and the unique Crassulacean acid metabolism (CAM) photosynthetic pathway (Chang et al. 2021). *Sedum* Linnaeus is a significant genus within the Crassulaceae, comprising approximately 470 species, predominantly occurring in the Northern Hemisphere, with a few species dispersed to Africa and South America in the Southern Hemisphere (Fu and Ohba 2001; ’t Hart and Bleij 2003; Thiede and Eggli 2007). However, *Sedum* is taxonomically challenging, with molecular phylogenetic studies revealing its highly polyphyletic nature (Carrillo-Reyes et al. 2009; Mort et al. 2010; Messerschmid et al. 2020). Specifically, *Sedum* species are dispersed across multiple clades in phylogenetic trees, with some occupying basal positions, indicating both a complex evolutionary history and the inadequacy of traditional morphological classification systems (Mort et al. 2001; Gontcharova et al. 2006). Recently, Messerschmid et al. (2020) proposed merging 14 previously independent genera into *Sedum*, as phylogenetic and morphological evidence demonstrated that these genera lacked clear monophyletic relationships and could not be distinguished by stable diagnostic characters. Intraspecific phenotypic plasticity and adaptive radiation further complicate *Sedum* taxonomy. For example, transplant experiments of *S. yangjifengensis* Z. W. Zhu, X. G. Le, L. F. Li, S. P. Chen & B. Chen (Zhu et al. 2023) revealed substantial morphological variation in flower stem numbers (ranging from solitary to hundreds per plant) under comparable climatic conditions. Similarly, nrITS and cpDNA lineage analyses of Taiwanese *Sedum* plants confirmed two independent migration lineages originating from Japan and mainland East Asia, each exhibiting parallel speciation (Ito et al. 2017). These findings, combined with frequent intergeneric hybridization in Crassulaceae (Uhl 1961), suggest that geographic isolation and ecological pressures may drive interspecific gene flow in *Sedum*, thereby blurring species boundaries. Although new species continue to be discovered (Wang and Xiong 2019; Ito et al. 2020; Zou et al. 2020, 2025; Huang et al. 2023; Chai et al. 2024), two taxonomic challenges persist: (1) specimen preparation of succulent tissues often distorts morphological structures (Meng et al. 2023), and (2) convergent evolution in vegetative and reproductive organs (Nikulin et al. 2016) complicates phenotypic delimitation among sympatric congeners. Thus, integrating traditional morphology with molecular phylogenetics is essential to resolve the taxonomic chaos within *Sedum*.

The Hunan Dong’an Shunhuang Mountain National Nature Reserve, located in the mid-section of the Yuechengling tectonic belt on the northwestern flank of the Nanling Mountains, lies within the biogeographic transition zone between the eastern margin of the Yunnan–Guizhou Plateau and the Jiangnan Hills. Its extensive mountainous terrain forms a unique ecotone blending features of the Nanling Montane ecosystem, the Yunnan–Guizhou Plateau, and subtropical evergreen broad-leaved forests. The complex topography generates distinct vertical climatic zonation, resulting in a flora marked by transitional and intermixed elements. This makes the reserve a critical yet understudied region for subtropical plant biogeography in China (Li 2020).

During a botanical survey in July 2024 at the reserve, a research team from Central South University of Forestry and Technology discovered two populations of an unknown *Sedum* taxon on a wet stone wall at an elevation of 650–1000 m in the core zone. This species exhibits sessile or subsessile flowers, carpels that are fused at the base, and awn-like arrangements at maturity. Its follicles are shallowly sac-shaped ventrally, aligning it morphologically with S. sect. Aizoon. Based on field observations and detailed morphological comparisons, we hypothesized that this plant might represent an undescribed species. In this study, we aimed to determine its phylogenetic position and taxonomic status through phylogenetic analysis and morphological comparisons. Integrated evidence from phylogenetic placement and morphological character comparisons establishes it as a new species.

## ﻿Materials and methods

### ﻿Morphological study

The morphology of the new species was investigated by systematically examining plant characteristics, integrating traditional herbarium research with modern digital resources. Specimens were examined using living collections and preserved materials from the herbaria CSFI, BNU, JIU, CCAU, and PE (abbreviations follow Thiers 2024). Digital resources, including the Chinese Virtual Herbarium (CVH) (https://www.cvh.ac.cn/) and the Plant Photo Bank of China (PPBC) (http://ppbc.iplant.cn/), were systematically reviewed to clarify morphological distinctions between the new species and its congeners.

### ﻿Phylogenetic analysis

DNA of the new species was extracted and PCR-amplified following the protocol described by Zhang et al. (2015). ITS sequences of 66 *Sedum* samples were downloaded from the National Center for Biotechnology Information (NCBI) (http://www.ncbi.nlm.nih.gov/), along with two sequences from *Rosularia* (Thiede and Eggli 2007; Messerschmid et al. 2020) (Crassulaceae) as the outgroup (GenBank accession numbers listed in Table 2). Two representative individuals from distinct populations were selected for molecular analysis: one from the Pangutang area of Shunhuang Mountain (X. W. Liao et al. SHS2154) and another from the Dalongjiang area of Shunhuang Mountain (Du Deng et al. SHS2155). Fresh leaves from these individuals were collected and preserved in silica gel within sealed plastic bags until use. Total DNA was extracted using a modified CTAB method (Doyle and Doyle 1987). The ITS region was amplified using PCR following Huang et al. (2021). For phylogenetic reconstruction, ITS sequences were aligned using MAFFT v. 7 (Katoh and Standley 2013) and manually adjusted in PhyloSuite (Zhang et al. 2020). The optimal substitution model (SYM+I+G4) for maximum likelihood (ML) was determined using ModelFinder (Kalyaanamoorthy et al. 2017) with the Akaike Information Criterion (AIC). ML trees were constructed with IQ-TREE (Nguyen et al. 2015; Hoang et al. 2018) using 1,000 replicates of the SH-approximate likelihood ratio test (SH-aLRT) and 1,000 ultrafast bootstrap (BS) replicates. Finally, the tree file was visualized using the online tool Interactive TVBOT (Xie et al. 2023).

## ﻿Results

### ﻿Morphological comparison

**Figure 1.**
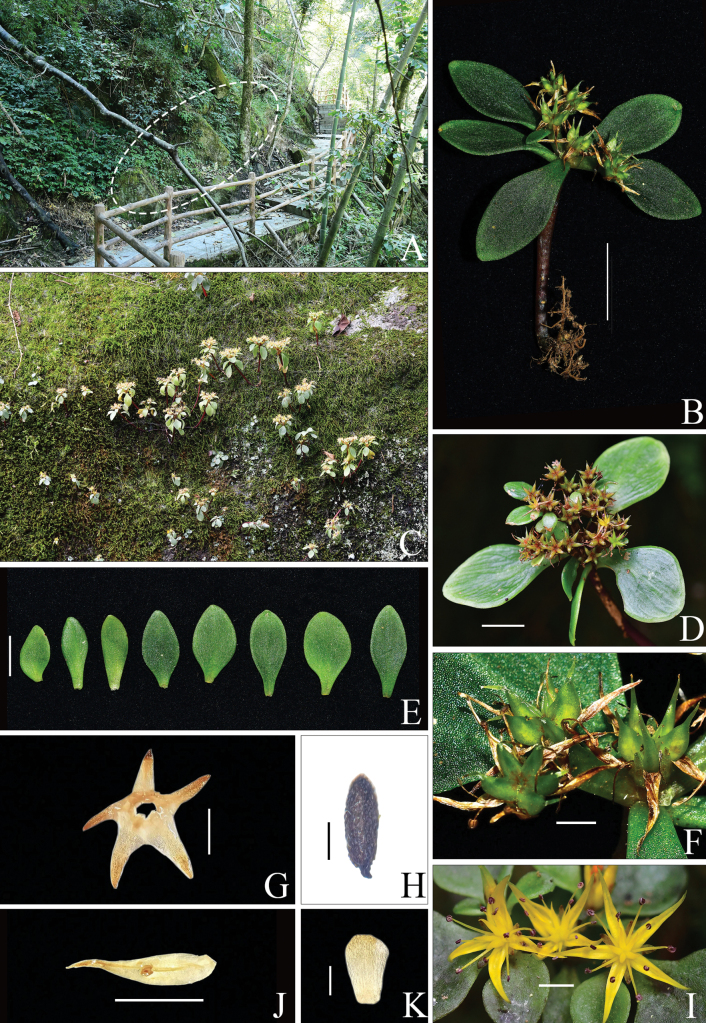
Morphology of *Sedum shunhuangense* L.Wu & Z.L.Feng, sp. nov. A. Habitat; B. Whole plant; C. Habitat; D. Front view of infructescence; E. Leaf transition; F. Lateral view of infructescence; G. Sepals; H. Seed; I. Flowers; J. Oppositipetalous stamen; K. Nectar scale. Scale bars: 1 cm (B, D, E); 0.5 cm (F, I, J); 1 mm (G); 0.3 mm (H); 0.1 mm (K). A, C, D, I. Photographed by Xiao Wen Liao (Pangutang area, voucher *SHS0908*); B, E, F, G, H, J, K. Photographed by Zi Lin Feng (Niulongqi area, voucher *SHS2154*).

In terms of plant morphology, the new species is a dwarf herb that grows on moss-covered rocks. Leaves are clustered at the stem apex, lacking basal leaves. The individual leaf shape is highly variable—narrowly triangular to elliptic—but all leaves are distinctly petiolate (Fig. 1E). The inflorescence is a lax cyme; flowers are yellow; sepals are subequal and connate at the base; petals are narrowly triangular with a mucronate apex; nectary scales are broadly cuneate; fruit carpels are slightly divergent; and seeds have a testa with distinct papillate ornamentation (Fig. 1F–K).

Although its overall plant habit and leaf shape show similarities to *S. alfredii* Hance and *S. yangjifengensis* (all three species lack basal leaves), *S. shunhuangense* possesses the following definitive diagnostic characters: (1) subequal sepals (vs. markedly unequal in both *S. alfredii* and *S. yangjifengensis*); (2) papillate seed coat (vs. smooth in *S. alfredii*); and (3) alternate leaves with caducous lower leaves during anthesis (vs. opposite leaves with persistent lower leaves in *S. yangjifengensis*). Several diagnostic traits distinguish between the three species (Table 1). Furthermore, we have compiled an identification key to the relevant species.

**Table 1.** Morphological comparison of *S. shunhuangense* and its allied species.

Traits		*S. shunhuangense*	*S. alfredii*	*S. wilsonii*	*S. yangjifengensis*	*S. lipingense*
Basal leaves		Absent	Absent	Absent	Absent	Present
Cauline leaves	Phyllotaxy	Alternate	Alternate	Alternate	Opposite or seldomly alternative on upper part	Alternate, sometimes opposite on lateral flowering stem
	Leaf state during flowering	Lower leaves deciduous	Lower leaves deciduous	Lower leaves deciduous	Lower leaves persistent	Lower leaves persistent
	Leaf shape	Narrowly triangular to elliptic	Linear-cuneate, spatulate, or obovate	Elliptic to obovate-spatulate	Suborbiculate or spatulate	Spatulate-obovate to spatulate-oblanceolate
	Spur at leaf base	Absent	Present	Absent	Present	Present
Apex leaves clustered as inflorescence bracts		Present	Absent	Absent	Present	Absent
Bract shape		Elliptic or obovate-spatulate	Linear-cuneate, spatulate, or obovate	Linear-subspatulate	Elliptic or obovate	Obliquely oblanceolate
Floral characters	Inflorescence	Corymbiform cyme	Corymbiform cyme	Corymbiform cyme	Corymbiform cyme	Scorpioid cyme
	Sepal shape	Narrowly triangular	Linear-spatulate	Ovate	Clavate or spatulate	Lanceolate-oblong
	Spur at sepal base	Absent	Present	Absent	Absent	Present
	Sepal size	Subequal, 0.7–1.5 × 0.5–0.8 mm	Unequal, 3–5 × 1–1.5 mm	Unequal, 3.5–4.5 × 1.5–1.8 mm	Unequal, 2.0–4.0 × 1.0–2.0 mm	Subequal, 2.5–3 × 0.5–0.8 mm
Appendages	Scale shape	Broadly cuneate	Spatulate-square	Square-subspatulate	Obtrapeziform	Broadly cuneate
	Seed coat ornamentation	Papillate	Smooth	Smooth	Papillate	Papillate

**Table 2.** Internal transcribed spacer (ITS) sequences of *Sedum* L. and *Rosularia* (DC.) Stapf (GenBank/NCBI), with taxon, accession, and voucher numbers, used for molecular analyses.

Taxon	Accession numbers	Voucher	Herbaria
**Outgroups**			
*Rosularia alpestris*	PV589526	*Qinghai-Tibet Plateau Scientific Expedition Team-Vegetation Research Group 12958*	PE
*Rosularia platyphylla*	KC988287	*Chen,L.Y., Zhao,S.Y. and Wang,Q.F. s.n*.	HIB
**Ingroups**			
*Sedum actinocarpum*	LC229263	*Ito1612*	TI
*Sedum alfredii*	FJ919948	*WUK267838*	WUK
*Sedum alfredii*	FJ919952	*IBK194562*	IBK
*Sedum alfredii*	AB930260	*Kokubugata 17191*	TI
*Sedum alfredii*	FJ919946	*WUK55069*	WUK
*Sedum alfredii*	AB930261	*Kokubugata 17192*	TI
*Sedum alfredii*	AB930259	*Kokubugata 17190*	TI
*Sedum alfredii*	FJ919950	*IBK194563*	IBK
*Sedum alfredii*	FJ919953	*WUK415208*	WUK
*Sedum alfredii*	FJ919947	*WUK208434*	WUK
*Sedum alfredii*	FJ919949	*IBK194564*	IBK
*Sedum alfredii*	FJ919951	*IBK114924*	IBK
*Sedum arisanense*	LC229272	*Ito1836*	TNS
*Sedum baileyi*	FJ919935	*LBG0064555*	LBG
*Sedum bergeri*	AY352897	Unavailable	Unavailable
*Sedum brachyrinchum*	LC229274	*Ito1359*	TI
*Sedum bulbiferum*	AB088628	*L. Niu 1999*	TI
*Sedum danjoense*	LC260127	*Ito3658*	TNS
*Sedum emarginatum*	LC530833	*Ito1062*	TNS
*Sedum erici-magnusii*	LC229235	*Ito2077*	TNS
*Sedum erythrospermum*	AB906473	*Tsutsumi 1504*	TI
*Sedum formosanum*	AB906474	*Kokubugata 11775*	TI
*Sedum hakonense*	AB930278	*Ito 623*	TI
*Sedum hangzhouense*	LC229236	*Ito2604*	TNS
*Sedum japonicum*	AB088617	*S. Mayuzumi, C00030*	TI
*Sedum jiulungshanense*	LC229243	*CMQ76*	TNS
*Sedum kawaraense*	LC731689	*Japan HK 4164*	TUS
*Sedum kawaraense*	LC731691	*Japan TI 7715*	TUS
*Sedum kiangnanense*	LC229244	*TI1030*	TNS
*Sedum lineare*	AB088623	*S. Mayuzumi, C00030*	TI
*Sedum lipingense*	MN150061	*ZRB1479*	IBK
*Sedum lungtsuanense*	LC260131	*Ito3563*	TNS
*Sedum makinoi*	AB088627	*S. Mayuzumi, C00086*	TI
*Sedum mexicanum*	AB088621	*S. Mayuzumi, C00001*	TI
*Sedum morrisonense*	AB906477	*Kokubugata 10831*	TI
*Sedum nagasakianum*	LC229249	*Ito2064*	TNS
*Sedum nanchuanense*	PV589525	*SHS1174*	PEY
*Sedum nanlingense*	MN105947	*MES06*	IBK
*Sedum nokoense*	AB906478	*Kokubugata 10426*	TI
*Sedum oligospermum*	PV589524	*Meng SY & Zhang JQ B087*	PEY
*Sedum onychopetalum*	KM111148	*130523nj67*	ACM
*Sedum oreades*	AB088632	*F. Miyamoto 9420140*	TI
*Sedum oryzifolium*	AB088618	*S. Mayuzumi, C00016*	TI
*Sedum polytrichoides*	KM111142	*130511hs21*	ACM
*Sedum rupifragum*	LC229254	*Ito2070*	TI
*Sedum sarmentosum*	AB088624	*S. Mayuzumi, C00008*	TI
*Sedum satumense*	LC229256	*Ito2295*	TI
*Sedum sekiteiense*	LC229295	*Ito1456*	TI
*Sedum shunhuangense*	PV589377	*SHS2154*	PEY
*Sedum shunhuangense*	PV589376	*SHS2155*	PEY
*Sedum spiralifolium*	KM111160	*130525sc03*	ACM
*Sedum subtile*	AB088622	*A. Shimizu 1999*	TI
*Sedum taiwanianum*	LC229297	*Ito2770*	TI
*Sedum tarokoense*	LC229298	*Ito2025*	TI
*Sedum tetractinum*	LC260135	*Ito3623*	TNS
*Sedum tianmushanense*	LC229261	*LP67*	TNS
*Sedum tosaense*	AB906488	*Kokubugata 16834*	TI
*Sedum triactina*	AB088629	*F. Miyamoto 9596091*	TI
*Sedum triangulosepalum*	LC229299	*Ito2508*	TNS
*Sedum tricarpum*	LC229259	*Ito2269*	TI
*Sedum trullipetalum*	AB088630	*F. Miyamoto 9420132*	TI
*Sedum truncastigmum*	LC229306	*Ito3254*	TNS
*Sedum wilsonii*	PV589527	*Meng SY & Zhang LG 019*	PEY
*Sedum yabeanum*	AB088626	*S. Mayuzumi, C00029*	TI
*Sedum yvesii*	PV589527	*Meng SY & Zhang LG 015*	PEY
*Sedum zentaro-tashiroi*	AB088619	*H. Ohba 1998*	TI

*S. shunhuangense* also exhibits some morphological similarities to *S. wilsonii* and *S. lipingense*; however, *S. shunhuangense* is distinguished by its elliptic to obovate-spatulate inflorescence bracts and leaves at the stem apex clustered at anthesis, lack of a rosette, and papillate seed coat. In contrast, *S. wilsonii* possesses elliptic to obovate-spatulate leaves, with distal leaves clustering at anthesis, and smooth seeds, while *S. lipingense* develops a conspicuous rosette and features obliquely oblanceolate inflorescence bracts.

In summary, *S. shunhuangense* exhibits significant differences from *S. alfredii*, *S. yangjifengensis*, *S. wilsonii*, and *S. lipingense* in sepal morphology, seed coat ornamentation, leaf arrangement and persistence, leaf clustering habit, presence/absence of rosette, and bract shape (see Table 1 for details).

### ﻿Molecular analysis

**Figure 2.**
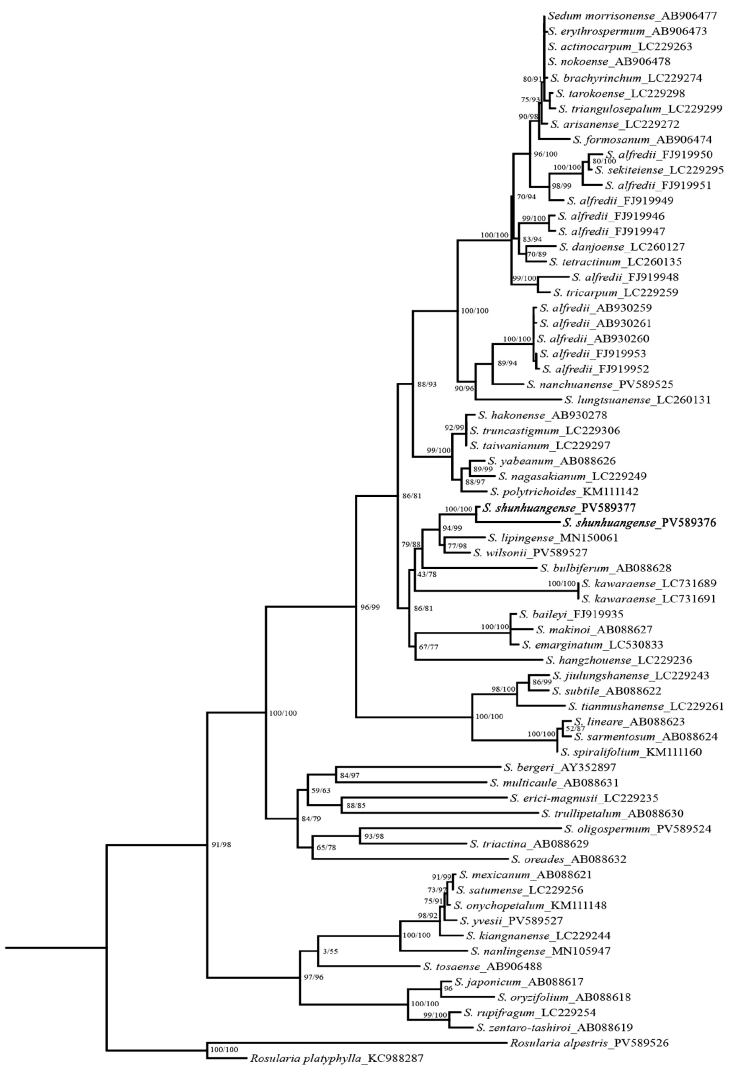
Maximum likelihood tree based on ITS sequences for Eastern Asian species of *Sedum*. Numbers near the nodes are the SHapproximate likelihood ratio test (left, SHaLRT) and ultrafast bootstrap (right, BS) support values. The new species is highlighted in bold.

We conducted phylogenetic analyses based on ITS sequences from 57 species (69 accessions) (Fig. 2). The aligned matrix comprised 628 nucleotide sites, including 238 conserved sites (37.90%), 308 parsimony-uninformative sites (49.04%), and 435 variable sites. ITS sequence alignment revealed high homology between *S. shunhuangense* and other *Sedum* species, strongly supporting its generic placement. Phylogenetic topology demonstrated that *S. shunhuangense* forms a highly supported monophyletic clade (SH-aLRT = 100%, BS = 100%) and is sister to *S. lipingense* and *S. wilsonii* (SH-aLRT = 94%, BS = 99%). These taxa are further nested within a clade containing *S. bulbiferum* (SH-aLRT = 79%, BS = 88%), collectively forming a distinct evolutionary lineage. Molecular data also indicate a considerable genetic distance between *S. shunhuangense* and *S. alfredii*, with no direct phylogenetic affinity observed.

## ﻿Discussion

We integrated morphological and molecular phylogenetic evidence to robustly support the recognition of *Sedum shunhuangense* as a distinct species within the genus *Sedum*. Phylogenetic analysis based on ITS sequences (Fig. 2) revealed that *S. shunhuangense* is sister to *S. lipingense* and *S. wilsonii* (SH-aLRT = 94%, BS = 99%), yet it is morphologically and genetically distinct from these closely related species.

Phylogenetic analysis of ITS sequences demonstrated that all *S. alfredii* samples from public databases (e.g., NCBI) form at least three independent monophyletic groups, indicating that *S. alfredii* represents a complex species complex requiring detailed systematic investigation. Notably, *S. shunhuangense* does not cluster with any *S. alfredii* clade but rather forms a sister lineage to the *S. alfredii* complex, with significant genetic divergence excluding intraspecific variation. This finding corroborates the complex evolutionary pattern characterized by the coexistence of phenotypic convergence and genetic divergence within the genus *Sedum* (Messerschmid et al. 2020).

## ﻿Taxonomic treatment

### ﻿ Sedum shunhuangense

Taxon classificationPlantaeSaxifragalesCrassulaceae

L.Wu & Z.L.Feng sp. nov.

3D6528C3-1AF7-5DA3-930B-405B1DF727C3

urn:lsid:ipni.org:names:77369128-1

Figs 1, 3, 4

#### Type.

China • Hunan, Yongzhou Don’an County, Shunhuang Mountain, 668 m; 26°27'32.09"N, 111°03'49.34"E, 21 July 2024, *X.W.Liao et al. SHS2154* (Holotype: CSFI!, Isotype: PEY!).

#### Diagnosis.

Phylogenetic analyses (Fig. 2) reveal that *S. shunhuangense* forms a sister group with *S. lipingense* and *S. wilsonii*. However, this species can be obviously distinguished from its allied species by the upper leaves aggregated at the flowering stem apex to form inflorescence bracts. Notably, *S. shunhuangense* shows distant phylogenetic relationships with *S. alfredii* and *S. yangjifengensis*; convergent evolution is observed in their phenotypic traits, particularly in inflorescence architecture and leaf morphology. However, it can be unequivocally distinguished by the following combination of characters (Table 1): (1) subequal sepals (vs. markedly unequal in both *S. alfredii* and *S. yangjifengensis*); (2) papillate seed coat (vs. smooth in *S. alfredii*); and (3) alternate leaves with caducous basal leaves during anthesis (vs. opposite leaves with persistent lower leaves in *S. yangjifengensis*).

**Figure 3.**
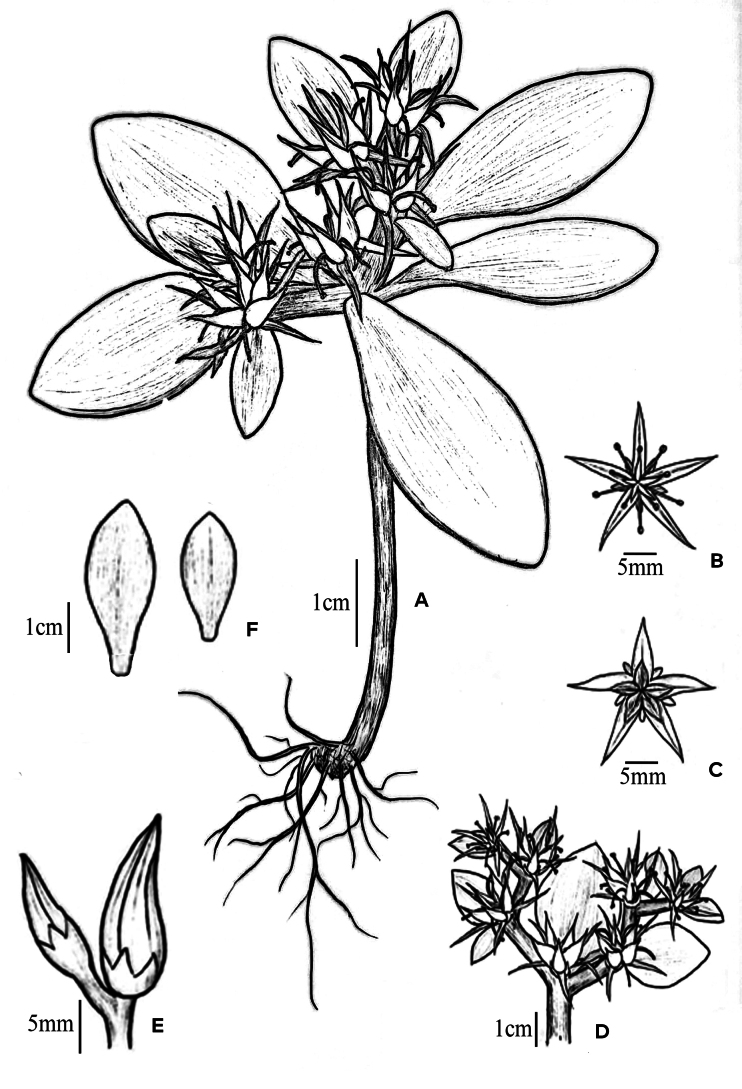
*Sedum shunhuangense* L.Wu & Z.L.Feng, sp. nov. A. Fruiting plant; B. Flower; C. Immature fruit; D. Infructescence; E. Flower bud; F. Leaf. Drawn by Bi-Shan Li.

#### Description.

Perennial succulent herb, glabrous throughout. Root system fibrous. Vegetative stems red, ascending, usually unbranched, 4.0–12.5 cm tall, 1.0–3.0 mm in diameter. Floral stems arising from apex or leaf axils of vegetative stems, basally unbranched, ascending, 0.6–4.5 cm tall, 0.5–1.5 mm in diameter. Leaves alternate, flat, slightly thickened; lacking basal leaves; lower leaves caducous at flowering, upper leaves 3–4 clustered at stem apex; leaf blade narrowly triangular to elliptic, spurless, petiolate, 0.9–2.5 cm long, 0.5–2.0 cm wide, apex obtuse, base attenuate, margin entire. Bracts leaf-like, borne at flower base, elliptic to obovate-spatulate, sessile, 4.0–8.0 mm long, 1.0–4.0 mm wide, apex acuminate, margin entire. Inflorescence lax corymbose-cymose, 2.5–6.0 cm wide; main axis reduced or absent, trichotomously branched from base into 3 subequal primary branches; each primary branch dichotomously divided apically into 2 secondary branches with scorpioid curvature, flowers alternate and secund, 1 per node. Flower sessile. Calyx 5-merous, subequal, basally connate, spurless, narrowly triangular, fleshy, flat, green, margin entire, 0.7–1.5 mm long, 0.5–0.8 mm wide, apex mucronate. Petals 5, free, pale yellow, narrowly triangular with a mucronate apex, 3.0–7.0 mm long, 1.2–1.5 mm wide, apex without appendages, base not constricted. Stamens 10, erect at anthesis, arranged in 2 whorls; antepetalous stamens 1.5–3.0 mm long, inserted slightly above petal base; antisepalous stamens 3.0–5.2 mm long; anthers black. Nectar scales 5, broadly cuneate, apex truncate, pale yellow. Style ca. 1.1 mm long. Carpels 3.0–4.0 mm long, erect, basally connate; ovules ca. 16 per locule. Follicles stellate-spreading at maturity, ca. 5.0 mm long, basally connate, ventrally carinate. Seeds numerous, ellipsoid, brown, 0.6 mm long, 0.3 mm wide; seed coat ornamentation papillate.

**Figure 4.**
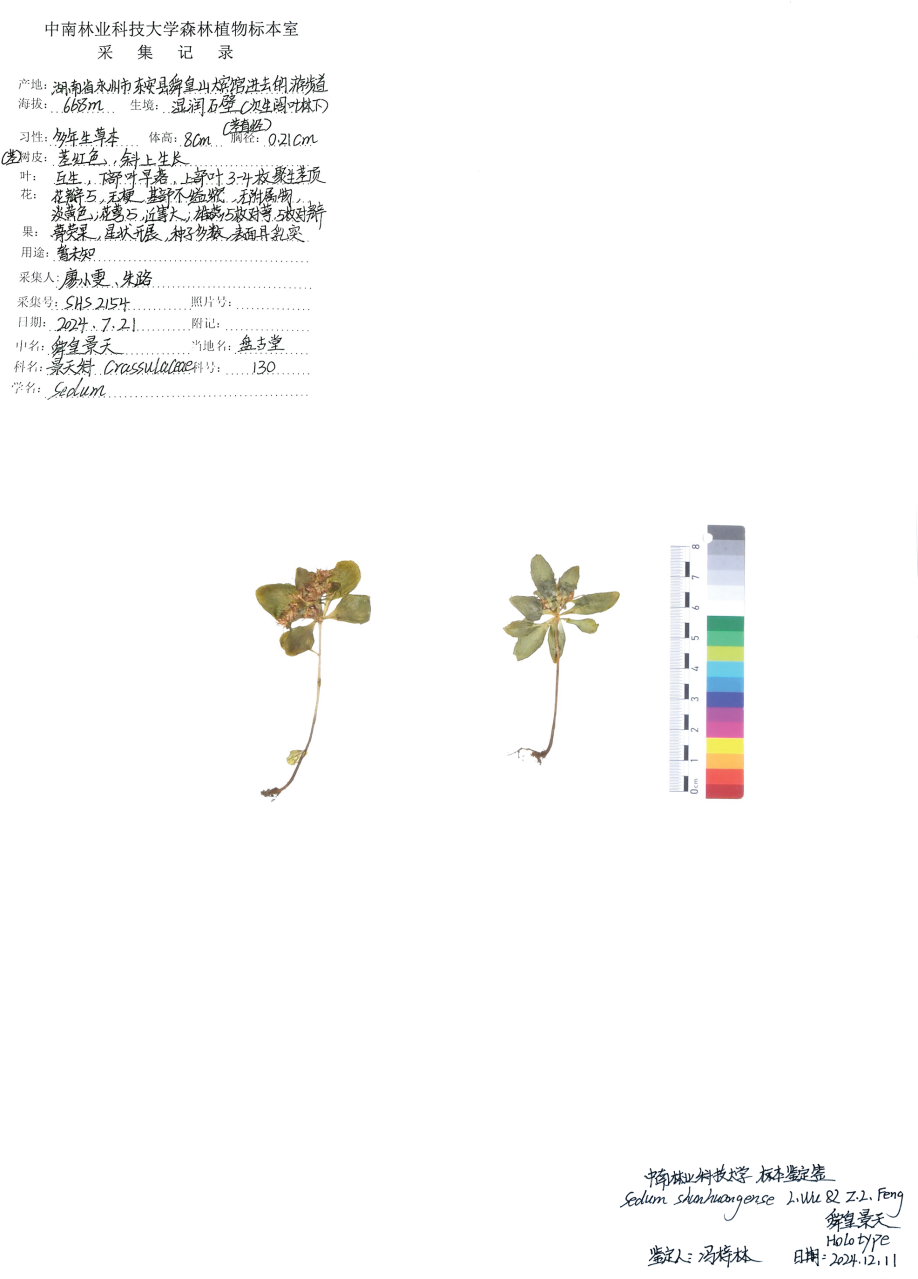
Holotype of *Sedum shunhuangense SHS2154* (CSFI).

#### Geographical distribution and habitat.

**Figure 5.**
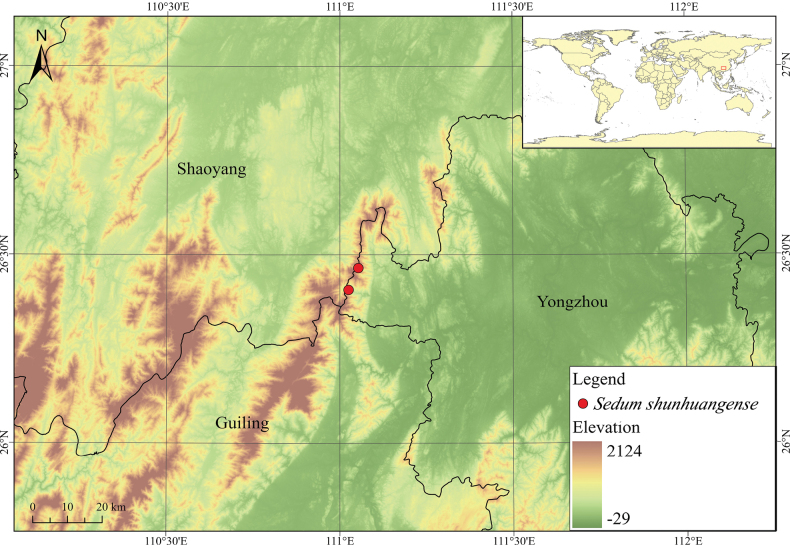
Distribution of *Sedum shunhuangense* L.Wu & Z.L.Feng in Hunan Province, China. Red circles indicate holotype and paratype localities.

Currently, *S. shunhuangense* is known only from damp limestone cliffs at elevations of 700–1000 m in the Shunhuangshan National Nature Reserve, Hunan Province, China (Fig. 5). This species usually lives on moss-covered rocks in secondary broad-leaved forests. Its deciduous lower leaves during anthesis may represent an adaptive strategy to optimize resource allocation in nutrient-poor rocky crevices, reflecting the genus’s phenotypic plasticity in heterogeneous habitats. The narrow distribution of this species suggests potential cryptic diversity or specialized adaptive mechanisms yet to be uncovered within the Nanling-Yunnan-Guizhou Plateau transitional zone. Therefore, it is urgent to carry out further investigations.

#### Phenology.

Flowering from June to July; fruiting from August to October.

#### Etymology.

*Sedum shunhuangense* L.Wu & Z.L.Feng, with the epithet derived from its type locality, Shunhuang Mountain. The nomenclature follows the International Code of Nomenclature for Algae, Fungi, and Plants (Turland et al. 2018), with holotype and paratype specimens permanently deposited.

#### Chinese name (assigned here).

shùn huáng jǐng tiān (舜皇景天).

#### Conservation status.

Comprehensive surveys within the protected area confirm that *S. shunhuangense* is strictly confined to limestone habitats on Shunhuang Mountain, with a total area of occupancy (AOO) of approximately 0.028 km^2^ (significantly below the 10 km^2^ threshold). The current population comprises approximately 200 mature individuals. Although one subpopulation adjacent to a tourist trail faces habitat degradation risks, the spatially proximal distribution of both subpopulations does not meet the fragmentation criterion. Consequently, Standard B is inapplicable. Under Criterion D of the IUCN Red List (IUCN 2023) (50 < total mature individuals < 250), this species is assessed as Endangered (EN).

#### Additional specimens examined (paratypes).

**China** • **Hunan**: Yongzhou County, Damiaokou Town, Pangutang, on moss-covered rocks in secondary broad-leaved forest, elevation 668 m, 26°24'03.71"N, 111°02'07.20"E, 13 July 2025, *Xiao-Wen Liao & Lu Zhu, SHS0908* (PEY); • Yongzhou County, Damiaokou Town, Niulongqi, 26°27'32.09"N, 111°03'49.34"E, 21 July 2024, *Xiao-Wen Liao & Lu Zhu, SHS2154* (CSFI).

### ﻿Diagnostic key of *S. shunhuangense* and related species

**Table:** 

1	Plants with conspicuous rosette leaves; bracts on inflorescence oblanceolate	***S. lipingense***
–	Plants without rosette leaves; bracts on inflorescence spatulate or elliptic	**2**
2	Sepals spurred at base	***S. alfredii***
–	Sepals not spurred at base	**3**
3	Leaves at stem apex not clustered at anthesis; seed coat smooth	***S. wilsonii***
–	Leaves at stem apex clustered at anthesis; seed coat papillate	**4**
4	Sterile stems present; leaf base spurred; sepals distinctly unequal	***S. yangjifengensis***
–	Sterile stems absent; leaf base not spurred; sepals subequal	***S. shunhuangense***

## Supplementary Material

XML Treatment for Sedum shunhuangense

## ﻿Additional information

### Use of AI

No use of AI was reported.

### Author ORCIDs

Zi-Lin Feng https://orcid.org/0009-0000-4283-5135

Xiao-Wen Liao https://orcid.org/0000-0003-2652-2862

Du Deng https://orcid.org/0009-0000-5476-4098

Long-Ping Tang https://orcid.org/0009-0003-6590-1491

Shi-Yong Meng https://orcid.org/0000-0002-6855-2990

Lei Wu https://orcid.org/0000-0003-1451-7855

### Data availability

All of the data that support the findings of this study are available in the main text.
